# Combined Effects of Test Media and Dietary Algae on the Toxicity of CuO and ZnO Nanoparticles to Freshwater Microcrustaceans *Daphnia magna* and *Heterocypris incongruens*: Food for Thought

**DOI:** 10.3390/nano9010023

**Published:** 2018-12-25

**Authors:** Marge Muna, Irina Blinova, Anne Kahru, Ivana Vinković Vrček, Barbara Pem, Kaja Orupõld, Margit Heinlaan

**Affiliations:** 1 Laboratory of Environmental Toxicology, National Institute of Chemical Physics and Biophysics, Akadeemia tee 23, 12618 Tallinn, Estonia; irina.blinova@kbfi.ee (I.B.); anne.kahru@kbfi.ee (A.K.); 2 Department of Materials and Environmental Technology, Tallinn University of Technology, Ehitajate tee 5, 19086 Tallinn, Estonia; 3 Estonian Academy of Sciences, Kohtu 6, 10130 Tallinn, Estonia; 4 Institute for Medical Research and Occupational Health, Ksaverska cesta 2, 10001 Zagreb, Croatia; ivinkovic@imi.hr (I.V.V.); bpem@imi.hr (B.P.); 5 Institute of Agricultural and Environmental Sciences, Estonian University of Life Sciences, Fr.R. Kreutzwaldi 5, 51006 Tartu, Estonia; kaja.orupold@emu.ee

**Keywords:** aquatic toxicology, nanomaterials, water flea, ostracod, zooplankton, feeding, natural waters

## Abstract

The chemical composition of the test medium as well as the presence of algae (microcrustaceans’ food) affects the bioavailability and thus the toxicity of metal nanoparticles (NP) to freshwater microcrustaceans. This study evaluated the effect of the addition of algae (*Rapidocelis subcapitata* at 7.5 × 10^6^ cells/mL) on the toxicity of CuO (primary size 22–25 nm) and ZnO NP (10–15 nm) to planktic *Daphnia magna* and benthic *Heterocypris incongruens* in artificial (mineral) and natural freshwater (lake water). The toxicity of ionic controls, CuSO_4_ and ZnSO_4_, was evaluated in parallel. When algae were added and the toxicity was tested in mineral medium, 48 h EC_50_ of CuO and ZnO NP to *D. magna* was ~2 mg metal/L and 6-day LC_50_ of *H. incongruens* was 1.1 mg metal/L for CuO and 0.36 mg metal/L for ZnO. The addition of algae to *D. magna* test medium mitigated the toxicity of CuO and ZnO NP 4–11-fold when the test was conducted in natural water but not in the artificial freshwater. The addition of algae mitigated the toxicity of CuSO_4_ (but not ZnSO_4_) to *D. magna* at least 3-fold, whatever the test medium. In the 6-day *H. incongruens* tests (all exposures included algae), only up to 2-fold differences in metal NP and salt toxicity between mineral and natural test media were observed. To add environmental relevance to NP hazard assessment for the freshwater ecosystem, toxicity tests could be conducted in natural water and organisms could be fed during the exposure.

## 1. Introduction

Nanoparticles (NP), defined as particles with at least one dimension in the range of 1–100 nm [1], may pose a hazard to biota when released into the environment. Assessing the environmental hazards of manufactured NP has been a real challenge for the scientific community due to the unique physicochemical properties of NP. Uncertainties regarding the NP behaviour during the toxicity testing and the questionable ecological relevance of respective experimental setups (e.g., unnatural test conditions and limited number of test species) of the standardised laboratory tests complicate the extrapolation of the laboratory test results to the real ecosystem [2,3]. As safety regulations for nanomaterials (consisting of ≥50% of NP) [1] are still under development, new knowledge on the effects of testing conditions on NP behaviour and toxicity is needed for the correct interpretation of the laboratory test results [4].

Compared to other traditional aquatic test species, microcrustaceans have been shown to be especially sensitive to metal-based NP [5]. However, the considerable variety of the toxicity values between different test species and also crustaceans species can be explained not only by species’ sensitivity pattern but partly or even predominantly by the test medium [2,6]. The chemical composition of the test medium and the feeding of aquatic test organisms during the exposure are the main factors which may affect metal bioavailability. ZnO and CuO NP reach the environment mainly due to their use in cosmetics, coatings, paints and pigments [7,8]. Modelling results suggest that ZnO NP pollution in some freshwater bodies may have already reached toxic concentrations while CuO NP pollution can impose localised hazards [9]. In freshwater, CuO and ZnO NP induce toxicity mostly via bioavailable toxic metal ions [5,10,11,12,13,14], causing ionic and osmoregulatory disturbances [13,15]. The dissolved organic matter in natural waters plays an important role in mitigating the toxic effects of not just metal ions [16,17,18], but also of metallic NP [19,20,21]. Water hardness is another important NP toxicity mitigator, promoting NP aggregation, decreasing dissolution, and allowing outcompeting of the metal ions at the biotic uptake sites [22,23,24].

Some standardised (sub)chronic microcrustacean toxicity tests (ISO 14371 [25], OECD 211 [26]) require adding high concentrations of algae in the test medium in order to feed the test organisms. Acute tests such as OECD 202 [27] often do not. As eutrophication and algal blooms are becoming more and more common in freshwater lakes [28], the addition of algae in the test medium helps to mimic environmental conditions. On the other hand, the toxicity results obtained in the presence of algae may not be valid for the periods outside of algal blooms, when algal concentrations may be up to 400 times lower compared to those used in OECD 211 tests [29]. ISO 14371 freshwater sediment toxicity testing with ostracods requires using even more elevated algal concentrations (7.5 × 10^6^ cells/mL) that exceed even the highest possible algal concentrations in nature [30,31].

This study compares the toxicity of two metal-based NP (CuO and ZnO) to planktic and benthic crustaceans. *Daphnia magna* is the most common aquatic invertebrate used for NP toxicity testing [5], while *Heterocypris incongruens* is a relatively novel alternative species to conventional sediment toxicity test organisms [32]. *H. incongruens* is especially relevant for the safety evaluation of NP, which may impose elevated risk to sediment biota by settling quickly in the waterbodies [9,33]. However, data on NP toxicity to benthic organisms are lacking [34,35]. The artificial freshwaters recommended for the standardised *D. magna* and *H. incongruens* toxicity tests have different ionic contents and do not contain dissolved organic matter. In order to add environmental relevance to the experiments, additional tests in natural waters with different organic matter contents were carried out. In addition, algae were added to some of the *D. magna* tests that normally do not require feeding the test organisms. The different combinations of the test media and the presence or absence of dietary algae in the toxicity experiments will give additional information on the environmental relevance of CuO and ZnO NP toxicity results from the laboratory tests. To our best knowledge, no studies have explored the combined effects of dietary algae and test media with different nutrient profiles or compared these effects for metal NP and respective soluble salts.

## 2. Materials and Methods

### 2.1. Chemicals

Nanoparticles used in this study were CuO NP (NNV-011; Intrinsiq Materials; powder form) and ZnO NP (NNV-003; Nanogate; powder form) obtained from the EU FP7 NanoValid project (“Developing of reference methods for hazard identification, risk assessment and LCA of engineered nanomaterials”, 2011−2015). CuSO_4_∙5H_2_O and ZnSO_4_∙7H_2_O (both Alfa Aesar) were used as ionic controls. CuO and ZnO NP stock suspensions (à 20 mL) were prepared at the concentration of 5 g metal/L in MQ water (MilliQ, >18 MΩ cm, Merck Millipore, Darmstadt, Germany) as has been previously described [36]. Stock suspensions were probe-sonicated for 4 min at 20 kHz (40 W) at continuous mode using 450 Ultrasonifier (Branson Ultrasonics Corporation, Danbury, CT, USA) after preparation and used for up to 4 weeks. The primary sizes of the CuO NP and ZnO NP (both uncoated) according to manufacturers’ data were 22−25 nm and 10−15 nm, respectively, which was in agreement with the TEM analysis showing 24.5 nm and 13.6 nm particle sizes.

### 2.2. Test Media

OECD 202 artificial freshwater (AFW) [27] and US EPA moderately hard reconstituted water (MHW) [37] were used as standard exposure media for *Daphnia magna* and *Heterocypris incongruens*, respectively. In addition, water from two Estonian lakes, Lake Ülemiste and Lake Raku, was used (Table 1). Lake Ülemiste is a natural eutrophic lake while Lake Raku is an artificial sandpit lake with a similar phosphorus concentration but lower dissolved organic carbon concentration and hardness. Lake water was collected between September and March and filtered through Millipore nitrocellulose filters (pore size 0.45 µm) and stored in the dark at 4 °C. The chemical analysis of lake waters was performed by an accredited laboratory (Tallinna Vesi Laboratories). The speciation of metal salts in test media was calculated using Visual MINTEQ version 3.1 [38].

### 2.3. Physico-Chemical Characterisation of Nanoparticle Suspensions

Dynamic light scattering (DLS) and electrophoretic light scattering (ELS) methodology was employed to measure the hydrodynamic diameter, zeta potential and polydispersity index of NP using ZetaSizer Nano ZS (Malvern Instruments, Malvern, UK) equipped with the 4.0 mW 633 nm laser (Model ZEN3600; 173° angle). NP samples (10 mg metal/L) were incubated for 0 h, 48 h, and 144 h at the toxicity test conditions and vortexed before the measurement.

The dissolution of NP was measured as initially described elsewhere [39]. Briefly, NP suspensions were prepared at 10 mg metal/L and incubated for 48 h (the duration of the *Daphnia magna* acute toxicity test) in OECD 202 AFW, Lake Raku water and Lake Ülemiste water with (7.5 × 10^6^ cells/mL) and without added algae. The samples were then ultracentrifuged at 362,769 × g for 30 min (duration of the whole cycle 60 min). For metal recovery control, salt solutions were always used in parallel. Metal concentrations in the supernatants were measured using graphite furnace atomic absorption spectroscopy (GF-AAS) analysis in the accredited laboratories of the Institute of Medical Research and Occupational Health (Zagreb, Croatia) and Estonian University of Life Sciences (Tartu, Estonia).

### 2.4. Test Formats of Bioassays

Three different experimental setups in three different test media were used to test the potential toxicity of NP and soluble salts (Table 1). The 48 h *Daphnia magna* acute immobilisation tests were carried out in accordance with the OECD 202 testing guidelines [27]. Briefly, *D. magna* neonates were pre-fed before the test and exposed to NP or metal salts at 21 °C in the dark. After 48 h of exposure, immobilised daphnids were counted. In addition to the standard *D. magna* test format, a modified one was applied, which included use of natural test media and/or addition of green algae *Raphidocelis subcapitata* at the concentration of 7.5 × 10^6^ cells/mL (the concentration required in the Ostracodtoxkit [30]). Tests were repeated 2 to 8 times with 2 to 4 technical replicates each including 5 daphnids. Based on the immobilisation results, 50% effect concentrations (EC_50_) were calculated.

Six-day subchronic *Heterocypris incongruens* toxicity testing was performed with a modified version of the OSTRACODTOXKIT F [30] (similar to ISO 14371 guidelines [25]) test procedure. Briefly, ostracod neonates (<24 h) were exposed to the NP or salts in the test media at 25 °C in the dark. Ostracods were pre-fed before the test and food was added to the test media (7.5 × 10^6^ cells of *R. subcapitata*/mL). After 6 days, mortality was recorded. Also, growth inhibition was calculated based on the length measurements under the dissection microscope (Olympus IMT-2, CellB software, Electro Optics, Cambridge, UK) before and after the incubation. As a modification, standard sand was not added to the test to exclude the metal adsorption on sand, which can significantly mitigate metal toxicity [40]. Each test was repeated 2 to 4 times with 2 technical replicates (10 ostracods in each) of all the tested concentrations to calculate EC_50_ values.

### 2.5. Statistical Analysis

MS Excel macro REGTOX [41] based on non-linear regression was used to calculate EC_50_ values. The “optimal” EC_50_ values were obtained from the log-normal model. Statistically significant differences between EC_50_ values were determined based on the 95% confidence intervals provided by the REGTOX program. The statistically significant differences between metal recovery results were also determined by the absence of overlap between the 95% confidence intervals.

## 3. Results

### 3.1. Behaviour of CuO and ZnO Nanoparticles in the Test Media

#### 3.1.1. Stability of Nanoparticle Suspensions

DLS and ELS data (Table S1) showed the low stability of both CuO and ZnO NP suspensions in all the test media (Table 2). Indeed, in lake water, the zeta potential (ζ) measured at the nominal concentration of 10 mg metal/L ranged from −15 to −19 mV, indicating that suspensions were relatively unstable. In both artificial freshwaters, suspensions were also unstable (ζ values ranged from −2 to −3.4 mV) [42]. 

The hydrodynamic diameters (D_h_) of CuO and ZnO NP as well as the polydispersity index (pdi) increased in time in all the test media, indicating intensive aggregation of NP during the exposure (Table S1). After 48 h incubation, the D_h_ values for CuO and ZnO NP were on average 4 times greater in artificial freshwaters compared to lake waters. Less intensive aggregation of the studied NP in organics-containing natural water probably occurred due to the NP-stabilising effect of dissolved organic matter (DOM) [43,44]. ZnO NP were more stable in Lake Ülemiste water (with higher DOM content) but the stability of CuO NP in both lake waters was similar. This may be explained by the counteraction of DOM concentration and water hardness [23,43,45], with both parameters being higher in Lake Ülemiste water.

Along with aggregation, sedimentation of NP has been shown to increase by high ionic strength and decrease by high organics content [45,46]. Phosphates have also been shown to stabilise CuO NP [47] but at higher concentrations than present in the natural waters of this study. A higher aggregation of NP in artificial waters compared to natural waters indicates the facilitated sedimentation of NP [44] in the former (Table S1). Sedimentation of algae also occurred in CuO and ZnO NP suspensions in mineral test medium but not in organics containing test medium (Figure S1). By contrast, algae in CuSO_4_ solution settled in all test media (slightly less in AFW) and algae in ZnSO_4_ solution showed comparable moderate sedimentation in all the test media.

Similarly to the homoaggregation of NP, the heteroagglomeration of NP and algae potentially occurred in artificial test media [19,48,49]. High ionic strength and low DOM concentrations can facilitate homoaggregation as well as heteroagglomeration [50,51,52]. Compared to dispersed NP, homoaggregated NP themselves enhance algae sedimentation [53], which at the same time is dependent on both algal [54] as well as on NP concentrations [50]. Both these parameters were high in the experimental setup, but the entrapment of algae by CuO NP at lower concentrations (2 mg/L) has also been previously shown [48].

#### 3.1.2. Dissolution of CuO and ZnO Nanoparticles in the Test Media

The dissolution of NP was evaluated by measuring levels of soluble metal forms released in the NP suspension (10 metal mg/L) after 48 h and 6 days of incubation in the test media (see Section 2.3). The total concentration of Zn and Cu in the supernatants, obtained after the ultracentrifugation of NP suspensions, represents the proportion of dissolved metal species (percentage of nominal concentration) in the test media (Table 3). The soluble forms of metal can be inorganic and organic complexes and free metal ions [56] (Table S2). The concentration of Cu dissolved from CuO NP was ≤2% of the nominal concentration in all the test media (Table 3), indicating a very low dissolution of CuO NP compared to ZnO NP. CuO NP dissolution is usually the highest in water characterised by the lowest pH, DOM and hardness values [47,57], but sometimes the link between these characteristics and dissolution is less straightforward [44]. The presence of algae and incubation duration did not have a significant effect on the CuO NP dissolution or Cu recovery from CuSO_4_ solutions. The Cu recovery from CuSO_4_ solutions was only 30−45% in all the test media (Table 3), indicating precipitation/adsorption as was discussed in our earlier work [58]. Visual MINTEQ modelling suggested the precipitation of Cu as tenorite in all the test media (Table S2), which implies that the release of copper ions may be underestimated due to speciation effects and subsequent metal recovery. Accordingly, a common term, “metal recovery”, will be used to refer to both metals recovered from NP dissolution experiments as well as from metal recovery control experiments (with metal salts) in the following discussion. Speciation effects were further demonstrated by the parallel analysis of metals in MQ water with the lowest pH value, which showed a significantly higher dissolution of CuO NP (6.9%) and Cu ion recovery (84%) from CuSO_4_ than in any of the exposure media (Table 3). The addition of algae (7.5 × 10^6^ cells of *R. subcapitata*/mL) increased the Cu recovery from CuSO_4_ by 3 to 12% in all test media after 48 h incubation. However, this increase was statistically significant only in lake waters (Table 3).

The dissolution of ZnO NP was 21−25% depending on the test media, while Zn recovery from Zn salt was 90−102% (Table 3) in all the media, and these values were comparable to Zn recovery in MQ water. ZnO NP dissolution has previously been shown to be high at a variety of ionic strength, pH, and DOM concentration values [57]. The presence of a high concentration of humic acids increases and high medium hardness decreases the ZnO NP dissolution [59]. However, we did not observe similar dissolution behaviour. The addition of algae increased the metal recovery from ZnO NP by 20−36% after 48 h of incubation, but the increase was significant only in AFW (Table 3). According to VisualMINTEQ modelling, the precipitation of Zn as hydrozincite occurred in all the test media (Table S2).

### 3.2. Toxicity of Cu and Zn Compounds to Daphnia magna and Heterocypris Incongruens

#### 3.2.1. Toxicity of Cu Compounds to *D. magna*

In *D. magna* acute toxicity tests without algae, lake waters significantly mitigated the toxicity of both CuO NP (up to 18-fold) and Cu salt (up to 4-fold) compared to AFW. The toxicity of Cu compounds (especially of CuO NP), was significantly lower in Lake Ülemiste than in Lake Raku water (Table 4). These results are in accordance with earlier published data on toxicity of other types of CuO NP and CuSO_4_ in natural waters with different DOM concentrations [20]. The direct link between Cu recovery in different media and toxicity was not revealed. This shows that not all dissolved copper species are equally bioavailable to microcrustaceans.

The addition of algae mitigated CuO NP toxicity in lake waters (5 to >10-fold) but not in AFW. Similar to the tests without algae, NP toxicity was lower in Lake Ülemiste water compared to Lake Raku water. The toxicity of Cu salt was mitigated more in AFW (8-fold) compared to lake waters (3-fold) by the addition of alga. Earlier studies have also shown that the addition of algae (*Chlorella*) to mineral test medium decreases Cu toxicity to a lower level than in organics-containing media (without algae) [60].

#### 3.2.2. Toxicity of Zn Compounds to *D. magna*

The effect of lake waters on the toxicity of ZnO NP and Zn salt to *D. magna* was quite different from Cu compounds: a 3‒4-fold increase in toxicity was observed compared to AFW in acute tests without algae (Table 4). Humic acids can increase ZnO NP toxicity to daphnids while fulvic acids [19] slightly mitigate Zn toxicity [61]. Dissolved organic matter also has much less affinity to Zn ions compared to Cu ions (Table S2). The water hardness, which mitigates Zn toxicity [22], was the highest in AFW, potentially explaining the higher Zn toxicity in natural water with lower hardness. Natural waters also had slightly higher pH which can increase Zn toxicity [61]. Despite the 4-fold difference in Zn recovery upon ultracentrifugation (in the absence of algae), toxicity of ZnO NP and ZnSO_4_ to *D. magna* was comparable. This may be explainable by the enhanced dissolution of the metal NP upon contact with the living cell [18].

The presence of algae slightly but significantly reduced the toxicity of Zn compounds to *D. magna* in lake waters, but did not change or even increased the toxicity (in case of ZnSO_4_) in AFW (Table 5). The toxicity of ZnSO_4_ was significantly higher in Lake Raku water compared to Lake Ülemiste water in the experiments with algae, possibly due to the difference in water hardness [59].

#### 3.2.3. Toxicity of Cu and Zn Compounds to *H. incongruens*

Similar to tests with *D. magna*, CuO NP were significantly less toxic than CuSO_4_ in the 6-day ostracod toxicity tests (Table 4), but there was only one case where toxicity was significantly affected by the test medium. The CuSO_4_ was up to 2-fold less toxic in Lake Ülemiste water compared to MHW and Lake Raku water, probably due to the highest DOM content being in Lake Ülemiste water (Table 2). Sublethal (mortality <20%) concentrations of CuSO_4_ enhanced the growth of ostracods (up to 41%) in all test media (Table S3).

As for Cu-compounds, the chemical composition of the test media had very little effect on the toxicity of Zn compounds to *H. incongruens* (Table 4). There were no statistically significant differences between the toxicity of ZnO NP and Zn salt. The toxicity of ZnO NP was significantly lower in Lake Ülemiste water compared to MHW, but no medium effect was observed for Zn salt. Sublethal concentrations of ZnSO_4_ increased the body length of ostracods by 17% in MHW (Table S3), probably due to the absence of Zn in this water.

Despite being a less common test organism compared to *D. magna*, the data on *H. incongruens* sensitivity to metals obtained in this study were consistent with those available in the literature. The previously published LC_50_ values for ZnSO_4_ and CuSO_4_ were in the range from 0.7 to 12 mg Zn/L and from <0.3 to 0.9 mg Cu/L, respectively, despite the fact that reference sediment with possible toxicity mitigating effect was applied [62,63]. Surprisingly, Zn toxicity was the lowest (LC_50_ 12 mg Zn/L) in distilled water as exposure medium [63].

## 4. Discussion

### 4.1. Combined Effect of the Media and Feeding on *D. magna* Toxicity Test Results

The effect of the addition of algae on the toxicity of copper and zinc compounds using modified formats of *D. magna* acute immobilisation testing (OECD 202) are summarised in Table 5. The toxicity mitigating effect of the added algae (as seen in both metal NP exposure in lake water and CuSO_4_ in all the test media) was anticipated, because feeding on organic compounds and the presence of extracellular polymeric substances of some algae have been shown to mitigate metal toxicity [64,65]. In addition, uncontaminated algae may help clear the gut of daphnids of metal NP [66]. The lack of effect and even the increased toxicity of the tested compounds in the presence of algae, as seen for CuO and ZnO NP in artificial freshwater and for Zn salt, was unexpected (Table 5).

The addition of algae could potentially change the toxicant exposure for the test organism. One possible explanation may be the internalisation of metals by algae, leading to foodborne metal exposure [62,66,67,68] especially in lake Raku water with lower ionic strength [22]. The concurrent sedimentation (or heteroagglomeration) (see 3.1.1) of NP and algae that was observed in AFW could have increased their simultaneous uptake due to daphnids turning to bottom-feeding. Almost no clear correlations were observed for toxicity and changes in the dissolution of either the metal NP or metal salt upon the addition of algae in the test medium (Table 5). As an exception, the absence of a mitigating effect of algae on ZnO NP toxicity in AFW can partly be due to the increased Zn recovery in the presence of algae (Table 5). Altogether, the effects of addition of algae cannot be considered analogous to the addition of dissolved organic matter in the test medium in case of CuO and ZnO NP in AFW.

The use of microalgae for feeding the crustaceans during NP exposure increases the environmental relevance of laboratory testing. The effect of algae concentrations on the interactions between NP and algae is yet to be determined, but high algal concentrations may lead to the heteroagglomeration of algae and NP. According to Stevenson et al. [29], using environmentally relevant algae concentrations in a chronic *Daphnia pulicaria* exposure to nano Ag significantly increased the adverse effects compared to the normal feeding rate recommended by the *D. magna* chronic toxicity test (OECD 211) guidelines.

### 4.2. Differences between CuO and ZnO Nanoparticle Toxicity to D. magna and H. incongruens

Compared to *D. magna* test with algae, the toxicity of Zn and Cu salts and ZnO NP to ostracod was only slightly higher in artificial freshwaters, but significantly higher (more than 30-fold) for CuO NP in lake waters (Table 4). The differences for Zn and Cu salts, ZnO NP and CuO NP in AFW may be explained by the 3-fold-longer test duration (Table 1) and the different bioavailability of metal species formed in the two different artificial test media (OECD AFW and US EPA MHW, see Table 2). For example, the toxicity of CuO and ZnO NP to *D. magna* has been shown to be higher in MHW compared to OECD AFW despite the higher dissolution in OECD AFW [69].

The negligible effect of the natural water on CuO toxicity to ostracod can be partially explained by the non-permanent nature of changes in metal bioavailability, induced by water parameters such as hardness [70,71] and the presence of humic acids [45]. For instance, the mitigative effects can last long enough to be evident in *D. magna* exposure (48 h) but not in *H. incongruens* exposure (6 days). The behaviour of the test organisms can also influence their exposure to toxicants. The planktic species *D. magna* is mostly exposed to soluble or suspended metal species, whereas benthic *H. incongruens* is more exposed to settled agglomerates of metal compounds. Agglomeration and sedimentation does not necessarily mean that metal compounds are less bioavailable to the test organisms [72]. Ostracods have been shown to be a more vulnerable organism to metal-polluted river sediment compared to water fleas despite the latter ones being more sensitive to metals in an exposure without sediment [73]. As concurrent sedimentation of NP and algae occurred in artificial freshwaters, daphnids may have turned to bottom feeding to access the settled algae. As a result, both daphnids and ostracods could have had similar exposure to the toxicants, feeding on settled NP aggregates along with settled algae. As algae remained suspended in natural waters, daphnids could feed in the upper layers of the test vessel, avoiding potentially higher metal NP concentrations on the bottom of the test vessel.

The effect of the addition of algae in *D. magna* and *H. incongruens* test medium cannot be directly compared in this study as *H. incongruens* toxicity tests without the addition of algae were not conducted to avoid starvation of the test organisms. Based on the literature, the effect of the addition of algae on toxicity of metals in organics-free test medium seems to be similar for both daphnids and ostracods despite the differences seen in natural waters for CuO as explained in the previous paragraph. Toxicity tests carried out for 48‒96 h with adult ostracods *Cypris subglobosa* and *Stenocypris major* in tap water and well water with no addition of algae (and no sediment) resulted in lower LC_50_ for Cu (0.025 to 0.055 mg Cu/L) and slightly to much higher LC_50_ for Zn (1.2 to 85 mg Zn/L) compared to the results obtained in this study, indicating the toxicity mitigating effect of algae on Cu and toxicity enhancing effects on Zn in artificial freshwater [35,74].

## 5. Conclusions

It is well known that the chemical composition of toxicological test media affects the bioavailability and thus the toxicity of metal nanoparticles (NP) to aquatic test organisms. However, it is poorly understood to what extent the addition of algae—food for microcrustaceans—into the test medium modulates the toxic effect. This aspect must be addressed since, in the (sub)chronic microcrustacean toxicity tests, algae are added as food by default. In this study, the combined effects of artificial versus natural test media and addition of algae (*Raphidocelis subcapitata* at 7.5 × 10^6^ cells/mL) on the toxicity of CuO and ZnO NP to planktic *Daphnia magna* and benthic *Heterocypris incongruens* was evaluated in standardised and modified test formats. Natural freshwater and addition of dietary algae in 48 h *D. magna* exposure were used as modifications.

- Subchronic (6 day) *H. incongruens* LC_50_ for CuO NP was 1.1 mg Cu/L, and 0.22 mg Cu/L for CuSO_4_, in US EPA mineral water. For both ZnO NP and ZnSO_4_, the respective 6-day LC_50_ was 0.36 mg Zn/L. For comparison, upon the addition of dietary algae in mineral medium, 48 h EC_50_ of CuO and ZnO NP for *D. magna* was ~2 mg metal/L;
- Compared to standard mineral media, natural freshwater mitigated CuO NP toxicity (4–18-fold) and increased ZnO NP toxicity (3–4-fold) for *D. magna*. For Cu and Zn salts, the toxicity change followed the same pattern with 3–4-fold mitigation and an increase in natural water. In *H. incongruens* tests (all including algae), toxicity was mitigated only up to 2-fold (CuO NP and Cu salt) or remained the same (Zn compounds) in natural water;
- Upon the addition of algae to *D. magna* for 48 h in OECD mineral medium, no toxicity mitigating effect was recorded for CuO NP, possibly due to the sedimentation of algae and NP. CuSO_4_ 48 h EC_50_, however, decreased 8-fold. For ZnO NP and ZnSO_4_, the added algae resulted in comparable or even increased (ZnSO_4_) toxicity;
- Algae in natural freshwater mitigated both CuO NP (5 to >10 fold) and Cu salt (3-fold) toxicity. The toxicity of ZnO was also significantly reduced (4-fold) but Zn salt toxicity remained unchanged.

According to the results of the modified *D. magna* and *H. incongurens* test formats, toxicity data from standardised acute/subchronic exposures may be overestimated for Cu-compounds and underestimated for Zn-compounds for eutrophic freshwaters during algal blooms. Also, our experiments once more demonstrated that the extrapolation of toxicity values obtained using planktic test species to other groups of aquatic microcrustaceans (e.g., benthic) may lead to significant mistakes in the environmental hazard evaluation, especially in the case of metal-based NP.

## Figures and Tables

**Table 1:** Experimental setup.

Experiment Type	Test Organism	Test Duration	Algae *R. subcapitata* (Cells/mL)	Test Medium
Acute	*Daphnia magna*	48 h	no	AFW, two lake waters
Acute	*Daphnia magna*	48 h	7.5 × 10^6^	AFW, two lake waters
Subchronic	*Heterocypris incongruens*	6 days	7.5 × 10^6^	MHW, two lake waters

AFW—OECD 202 artificial freshwater; MHW—US EPA moderately hard reconstituted water.

**Table 2:** Chemical composition of the test media. Hardness values for artificial freshwaters were calculated based on Ca^2+^ and Mg^2+^ concentrations. Water was collected twice from Lake Raku and 4 times from Lake Ülemiste. The mean (SD) of the parameters of lake waters, collected at different times, is given. Conductivity in AFW and MHW was measured; other values were calculated based on the ionic composition.

	*D. magna* AFW	*H. incongruens* MHW	Lake Raku	Lake Ülemiste
pH	7.8	7.6	8.3 (0.035)	8.2 (0.45)
Conductivity 25 °C (μS/cm)	640 ^1^	343 ^2^	283 (5.7)	399 (60)
Total organic carbon (mg/L)	0	0	5.1 (0.21)	10 (0.45)
Total hardness (mg-ekv/L)	5	1.7	2.7 (0.10)	3.9 (0.56)
Total phosphorous (mgP/L)	0	0	0.035 (0.00071)	0.030 (0.012)
Total nitrogen (mgN/L)	0	0	0.62 (0.13)	1.4 (0.40)
Cl^−^ (mg/L)	73	1.9	3.4 (0.28)	11 (2.1)
SO_4_^2−^ (mg/L)	48	93	22 (0)	29 (4.5)
Ca^2+^ (mg/L)	80	14	44 (2.5)	66 (11)
Mg^2+^ (mg/L)	12	12	4.6 (0.10)	7.8 (0.50)
Na^+^ (mg/L)	18	26	2.7 (0.021)	6.7 (1.0)
Cu^2+^ (μg/L)	0	0	1.0 (0.25)	0.64 (0.19)
Zn^2+^ (μg/L)	0	0	0.66 (0.45)	0.69 (0.36)

MHW—US EPA moderately hard reconstituted water; AFW—OECD 202 artificial freshwater; ^1^ value from [55]; ^2^ measured using ZetaSizer Nano ZS (Malvern Instruments, UK).

**Table 3:** Metal recovery (%) from metal nanoparticles and salt upon ultracentrifugation after 24 h, 48 h or 6 days of incubation of test media at 10 mg metal/L without the test organisms. The addition of algae *Raphidocelis subcapitata* (7.5∙× 10^6^ cells/mL) was used in part of the analysed samples. The mean (standard deviation) based on 1‒2 experiments is presented.

	MQ	AFW		MHW			Lake Raku			Lake Ülemiste		
Incubation Time	24 h	48 h		48 h ^1^		6 Day ^1^	48 h		6 Day ^1^	48 h		6 Day ^1^
Algae	No	No	Yes	No	Yes	Yes	No	Yes	Yes	No	Yes	Yes
CuO	6.9 (1.6)	0.67 ^2^ (0.47)	1.5 (0.8)	0.42	1.7	1.8	0.90 ^2^ (0.42)	1.1 (0.53)	1.0	1.2 ^2^ (0.58)	1.8 (0.29)	2.0
CuSO_4_	84 (5.6)	37 (6.2)	47 (4.9)	37	40	26	33 (0.76)	45 (4.1)	31	32 (0.81)	42 (1.3)	37
ZnO	27 (1.9)	24 (6.3)	44 (6.8)	25	55	51	21 (2.7)	57 (37)	83	23 (1.5)	54 (28)	76
ZnSO_4_	88 (9.1)	102 (14)	94 (5.3)	97	97	81	90 (0.012)	86 (19)	97	91 (2.0)	90 (1.3)	90

MQ—Milli-Q water; AFW—OECD 202 artificial freshwater; MHW—US EPA moderately hard reconstituted water; ^1^ one experiment was conducted; ^2^ the values include previously published data [55,58].

**Table 4:** Acute and subchronic toxicity of different metal formulations to *Daphnia magna* and *Heterocypris incongruens* based on nominal concentrations. Data are presented as E(L)C_50_ (95% confidence interval), mg metal/L based on REGTOX “optimal” model. N = 2–8 (*D. magna*) and n = 2–4 (*H. incongruens*).

	*D. magna* Acute EC_50_ (48 h)			*D. magna* Acute EC_50_ (48 h) with Algae			*H. incongruens* Subchronic LC_50_ (6 days) with Algae		
	AFW	Lake Raku	Lake Ülemiste	AFW	Lake Raku	Lake Ülemiste	MHW	Lake Raku	Lake Ülemiste
CuO NP	1.6 * (1.1−3.4)	6.3 (3.9−13)	28 (18−53)	2.0 (1.7−2.2)	68 (57−80)	>150	1.1 (1.1−1.6)	1.9 (1.4−3.3)	2.2 (0.77−4.2)
CuSO_4_	0.053 * (0.047−0.059)	0.15 (0.089−0.18)	0.22 (0.20−0.25)	0.41 (0.35−0.51)	0.50 (0.35−0.70)	0.65 (0.56−0.76)	0.22 (0.20−0.24)	0.25 (0.23−0.25)	0.44 (0.42−0.49)
ZnO NP	1.9 * (1.7−2.2)	0.50 (0.46−0.58)	0.71 (0.59−0.97)	1.7 (1.6−2.2)	1.9 (1.4−2.6)	3.1 (2.1−4.5)	0.36 (0.30−0.49)	0.51 (0.38−0.62)	0.65 (0.54−0.70)
ZnSO_4_	2.3 * (1.9−2.9)	0.59 (0.53−0.79)	0.76 (0.66−0.91)	1.5 (1.4−1.7)	0.84 (0.82−0.89)	1.3 (1.3−1.4)	0.36 (0.12−0.46)	0.43 (0.39−0.48)	0.43 (0.40−0.50)

EC_50_—concentration immobilising 50% of test organisms; LC_50_—concentration lethal to 50% of test organisms; NP—nanoparticles; AFW—OECD 202 artificial freshwater; MHW—US EPA moderately hard reconstituted water; * The calculation of these values included previously published data [36].

**Table 5:** The effect of addition of algae on the toxicity of copper (CuO NP and CuSO_4_) and zinc (ZnO NP and ZnSO_4_) compounds in 48 h *D. magna* acute immobilisation assay and on the metal recovery (reflects dissolution for NP). Background colour coding is explained below the table and shows statistically significant effects.

		Copper Compounds			Zinc Compounds		
		AFW	Lake Raku	Lake Ülemiste	AFW	Lake Raku	Lake Ülemiste
Change in toxicity ^1^ (EC_50_ with algae/EC_50_ no algae)	NP	1.3	11	>5	0.9	3.8	4.4
	salt	7.7	3.3	3.0	0.65	1.4	1.7
Change in metal recovery ^2^ (no algae/with algae)	NP	0.45	0.82	0.67	0.55	0.37	0.43
	salt	0.79	0.73	0.76	1.1	1.0	1.0
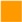 increase 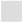 no effect 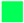 ≤5 fold decrease 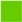 ≥5 fold decrease							

^1^ calculation based on data in Table 4. ^2^ calculation based on data in Table 3. AFW—OECD202 artificial freshwater; NP—nanoparticles.

## Supplementary Materials

The following are available online at http://www.mdpi.com/2079-4991/9/1/23/s1, Table S1: Characterisation of CuO and ZnO nanoparticle suspensions at 0 h, 48 h, and 6 days in five different test media. Table S2: Percentage of soluble and solid fraction predicted by Visual MINTEQ simulation results. Tabel S3. Change in *H. incongruens* growth (%) at the end of the 6-day experiment at sublethal concentrations. Figure S1: Examples of typical sedimentation of algae after 6 days of incubation with CuO and ZnO NP and respective soluble salts at 10 mg metal/L.
