# Supplementary material for: Combined Effects of Test Media and Dietary Algae on the Toxicity of CuO and ZnO Nanoparticles to Freshwater Microcrustaceans Daphnia magna and Heterocypris incongruens: Food for Thought
Source: Nanomaterials (Basel). 2018 Dec 25;9(1):23. doi: 10.3390/nano9010023 (PMC6359263; doi:10.3390/nano9010023)
Supplement: Supplementary file 1 [file nanomaterials-09-00023-s001.pdf]

Supplementary Information

Combined Effects of Test Media and Dietary Algae on the Toxicity of CuO and ZnO Nanoparticles to Freshwater Microcrustaceans *Daphnia magna* and *Heterocypris incongruens*: Food for Thought

Marge Muna [1,2,*], Irina Blinova [1], Anne Kahru [1,3], Ivana Vinković Vrček [4], Barbara Pem [4], Kaja Orupõld [5] and Margit Heinlaan [1,*]

[1] Laboratory of Environmental Toxicology, National Institute of Chemical Physics and Biophysics, Akadeemia tee 23, Tallinn 12618, Estonia; irina.blinova@kbfi.ee (I.B.); anne.kahru@kbfi.ee (A.K.)

[2] Department of Materials and Environmental Technology, Tallinn University of Technology, Ehitajate tee 5, Tallinn 19086, Estonia

[3] Estonian Academy of Sciences, Kohtu 6, Tallinn 10130, Estonia

[4] Institute for Medical Research and Occupational Health, Ksaverska cesta 2, Zagreb 10001, Croatia; ivinkovic@imi.hr (I.V.V.); bpem@imi.hr (B.P.)

[5] Institute of Agricultural and Environmental Sciences, Estonian University of Life Sciences, Fr.R. Kreutzwaldi 5, Tartu 51006, Estonia; kaja.orupold@emu.ee

* Correspondence: marge.muna@kbfi.ee (M.M.); margit.heinlaan@kbfi.ee (M.H.); Tel.: +372-639-8361 (M.M. & M.H.)

Table S1. Characterisation of CuO and ZnO NP suspensions at 0 h, 48 h, and 6 days in five different test media. Nominal concentration was 10 mg metal/L. The mean (SD) of 3 technical replicates of one experiment is given. NP suspension may be characterised as highly unstable if ζ values are in range of ±0–10 mV; relatively stable for ±10–20 mV; moderately stable for ±20–30 mV, and highly stable for values >±30 mV [40].

		MQ			AFW			MHW			Lake Raku			Lake Ülemiste		
	**Time (h)**	**0**	**48**	**144**	**0**	**48**	**144**	**0**	**48**	**144**	**0**	**48**	**144**	**0**	**48**	**144**
CuO	D_h (nm)	**207** (14)	**236** (8.9)	**198** (3.9)	**573** (41)	**1826** * (346)	**2142** * (848)	**565** (41)	**1638** * (302)	**2645** * (841)	**197** (10)	**393** (8.9)	**449** (18)	**335** (14)	**440** (20)	**452** (6.5)
	ζ (mV)	**−15** (0.15)	**12** (1.3)	**23** (0.46)	**−7.3** (2.0)	**−4.6** (0.59)	**−3.5** (0.37)	**−13** (0.7)	**−8.4** (1.2)	**−13** (4.0)	**−19** (0.44)	**−18** (0.17)	**−17** (0.36)	**−18** (0.25)	**−17** (0.46)	**−17** (0.55)
	pdi	**0.17** (0.062)	**0.21** (0.015)	**0.24** (0.0040)	**0.22** (0.045)	**0.78** (0.023)	**0.81** (0.12)	**0.24** (0.015)	**0.77** (0.23)	**1** (0.0)	**0.19** (0.010)	**0.28** (0.016)	**0.35** (0.017)	**0.25** (0.016)	**0.26** (0.018)	**0.35** (0.0065)
ZnO	D_h (nm)	**116** (2.5)	**337** (121)	**NS**	**639** (94)	**3855** * (1807)	**3871** * (2556)	**1225** (27)	**2846** * (711)	**3385** * (310)	**283** (50)	**1135** (55)	**1052** * (119)	**177** (4.6)	**609** (27)	**445** (17)
	ζ (mV)	**18** (0.42)	**−10** (1.5)	**NS**	**3.4** (0.40)	**−4.1** (0.14)	**−5.5** (0.52)	**−5.3** (0.29)	**−2.0** (0.11)	**−5.0** (0.30)	**−17** (0.56)	**−15** (0.25)	**−15** (0.49)	**−16** (1.3)	**−16** (0.92)	**−16** (0.21)
	pdi	**0.20** (0.019)	**0.53** (0.14)	**NS**	**0.34** (0.017)	**0.86** (0.25)	**0.84** (0.28)	**0.45** (0.049)	**0.60** (0.14)	**0.30** (0.13)	**0.40** (0.016)	**0.56** (0.11)	**0.46** (0.036)	**0.34** (0.015)	**0.38** (0.023)	**0.37** (0.029)

AFW: OECD202 artificial freshwater; MHRW: US EPA moderately hard reconstituted water; D_h: Hydrodynamic diameter; ζ: Zeta potential; pdi: Polydispersity index; NS: DLS analysis found the sample unsuitable for particle analysis; *: Data did not meet the DLS analysis quality criteria (particle concentration was low etc.).

Table S2. Speciation of metals in different test media predicted by Visual MINTEQ simulation.

	Test medium	pH [1]	Dissolved fraction (%)			Precipitated fraction (%)	
			Inorganic		Bound to DOM	Fraction	Type of mineral
			Free ion	Bound ion			
$CuSO_4$	MQ	4.9	97	2.7	0	0	
	AFW	7.5	0.080	0.12	0	100	Tenorite (CuO)
	MHW	7.7	0.021	0.093	0	100	Tenorite (CuO)
	Raku	7.9	0.0077	0.082	1.0	99	Tenorite (CuO)
	Ülemiste	8.3	0.0016	0.077	2.0	98	Tenorite (CuO), Covellite (CuS)
$ZnSO_4$	MQ	6.4	97	2.6	0	0	Hydrozincite
	AFW	7.4	52	4.5	0	43	Hydrozincite
	MHW	7.5	52	8.3	0	40	Hydrozincite
	Raku	8.1	3.3	1.3	0.071	95	Hydrozincite, Smithsonite, Zn-Al layered double hydroxide
	Ülemiste	8.4	0.47	1.3	0.016	99	Hydrozincite

[1] recorded after 6 days of incubation at 10 mg metal/L at 20 °C; AFW: OECD202 artificial freshwater; MHW: US EPA moderately hard reconstituted water; Ülemiste: water from lake Ülemiste; Raku: water from lake Raku.

Tabel S3. Change in *H. incongruens* growth (%) at the end of the 6-day experiment at sublethal concentrations. Negative values show growth inhibition and positive values growth enhancement in comparison to control organisms. Each value is based on measurements of 5 to 12 organisms from one exposure concentration. The mean (SD) of 2 experiments is given when possible.

	Test concentration (mg metal/L)	MHW	Raku	Ülemiste
CuO NP	0.1	−9.2	−5.6	1.2
	1	−4.2	6.3	−10
$CuSO_4$	0.05		27	
	0.1	41	−23	13
	0.2	4.1		
	0.25			9.8 (7.4)
	0.38			−1.9
ZnO NP	0.1	−9.8 (0.35)		
	0.25		−39	−20
	0.5		−47	−40
$ZnSO_4$	0.01		3.9	−8.6
	0.05		0.7	−17
	0.1	17	−1.7	−21
	0.25		−53	−39
	0.5	−41		

MHW: US EPA moderately hard reconstituted water; Ülemiste: water from lake Ülemiste; Raku: water from lake Raku; NP: nanoparticles.

CuO NP **$CuSO_4$** **ZnO NP** **$ZnSO_4$**

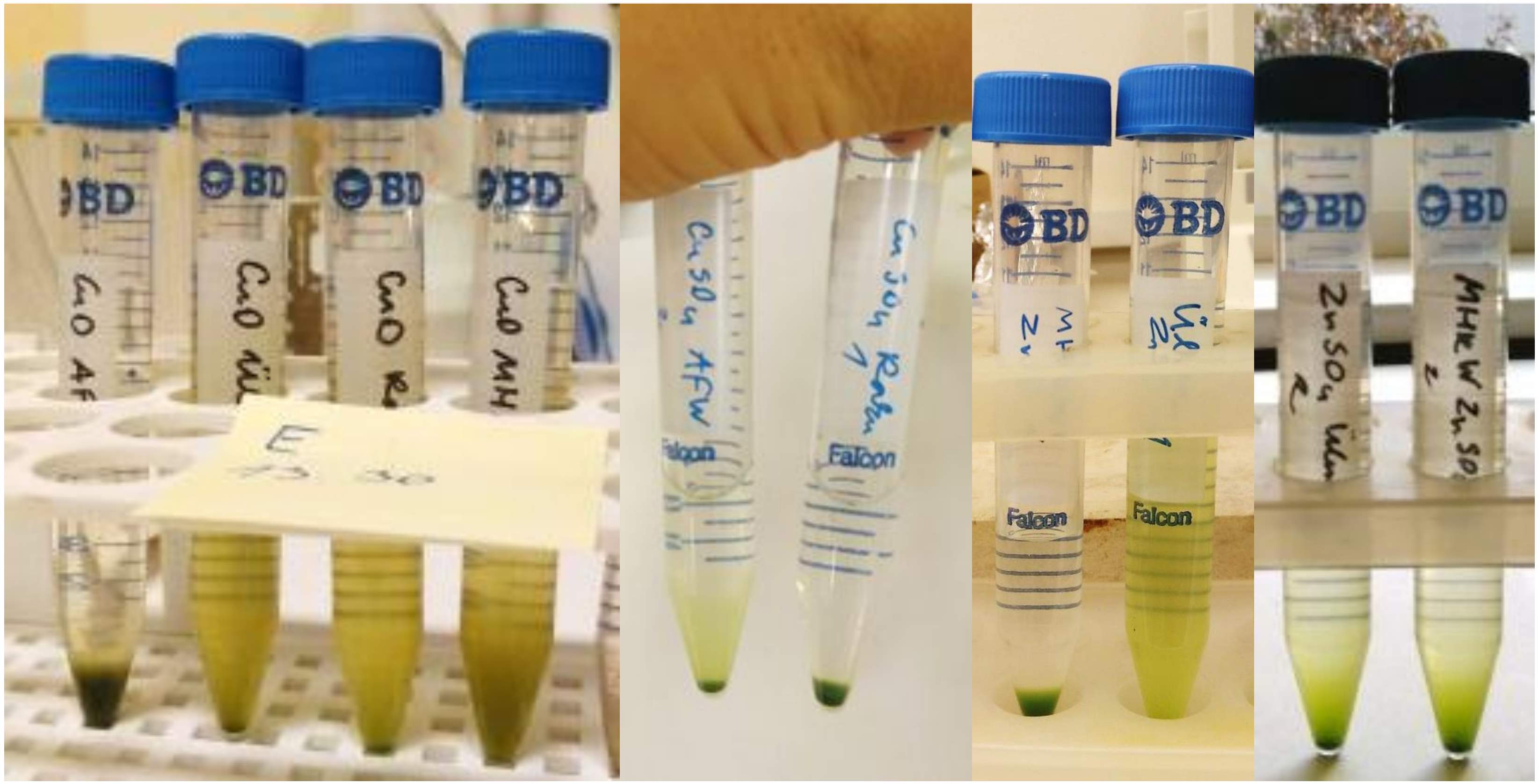

AFW **Ülemiste** **Raku** **MHW** **AFW** **Raku** **MHW** **Ülemiste** **Ülemiste** **MHW**

Figure S1. Examples of typical sedimentation of algae after 6 days of incubation with CuO and ZnO NP and respective soluble salts at 10 mg metal/L. MHW: US EPA moderately hard water; AFW: OECD 202 artificial freshwater; Ülemiste: water from lake Ülemiste; Raku: water from lake Raku.
